# Nutrition Indicators in Type 2 Diabetes Mellitus—Retrospective Study

**DOI:** 10.3390/biomedicines13051137

**Published:** 2025-05-08

**Authors:** Jakub Piersa, Wiktoria Bajek, Aleksandra Pilśniak, Agnieszka Jarosińska, Marta Pietrukaniec, Michał Holecki

**Affiliations:** 1 Student Scientific Society at the Department of Internal, Autoimmune and Metabolic Diseases, School of Medicine, Medical University of Silesia, 40-752 Katowice, Poland; s81252@365.sum.edu.pl (J.P.); victoriabajek@gmail.com (W.B.); 2 Department of Internal, Autoimmune and Metabolic Diseases, School of Medicine, Medical University of Silesia, 40-752 Katowice, Poland; ajarosinska@sum.edu.pl (A.J.); martpe@tlen.pl (M.P.)

**Keywords:** diabetes mellitus type 2, malnutrition, nutrition indicators, CONUT, PNI

## Abstract

**Background/Objectives**: This study aims to evaluate the prevalence and degree of malnutrition among patients with type 2 diabetes mellitus using the CONUT and PNI scores. **Hypothesis**: The CONUT and PNI scores provide a reliable assessment of the nutritional status of patients with type 2 diabetes. **Methods**: The retrospective study was run at the Department of Internal, Autoimmune and Metabolic Diseases in the Central Clinical Hospital of the Medical University of Silesia in Katowice from January to December of 2022. From 266 patients diagnosed with diabetes, only 64 met the criteria and were included in this study. **Results**: We found varying degrees of malnutrition among patients. Only 20.3% of them were well nourished. Mild to moderate malnutrition was observed in, altogether, 67.2% of patients. **Conclusions**: The strong correlation between the CONUT and PNI (r = −0.88) indices confirms their diagnostic value. The introduction of the CONUT or PNI tools into routine practice should be considered in patients with T2DM, especially those over 65 years of age, but taking into account the significant limitations of these indices and the influence of various factors on the laboratory data considered.

## 1. Introduction

Diabetes mellitus refers to a group of heterogeneous metabolic disorders characterized by hyperglycemia. It can be caused by impaired insulin secretion, impaired insulin action, or both. According to the WHO, in 2017, over 9 million people were suffering from type 1 diabetes, while 95% of people diagnosed with diabetes have type 2 diabetes. Over the last 3 decades, the prevalence of type 2 diabetes has increased dramatically in countries of all income levels. Today, approximately 537 million people around the world have diabetes. Most of them live in low- and middle-income countries.

Type 2 diabetes mellitus (T2DM) (non-insulin-dependent diabetes) can take various forms, from relative insulin deficiency to a major secretory defect with insulin resistance [1,2]. There are well-known risk factors for the development of type 2 diabetes. The two best known are obesity and lack of physical inactivity. Recent work shows differences between gender in risk factors, pathophysiology and complications. In men, T2DM is more often diagnosed at a lower age and with a lower body mass index, while obesity—the most important risk factor—is more common in women. Changing bad habits and promoting a healthy lifestyle play a key role in the prevention of DMT2 [3,4].

Chronically high glucose levels lead to nephropathy, neuropathy, retinopathy, and heart and blood vessel diseases [5].

In this study, we focused on T2DM, which is associated with insulin resistance in body tissues. Inflammation in the excess adipose tissue of obese patients leads to the secretion of adipokines, which increase the insulin resistance of cells, and lead to the disruption of transmembrane glucose transport [6].

In non-insulin-dependent types of diabetes, insulin resistance may be the cause of patient malnutrition, due to complications or nutritional errors. These problems can cause a low lymphocyte count and a weakened immune system, low albumin levels, swelling, low cholesterol levels, body weakness, dizziness, recurrent infections and also mental disorders.

As malnutrition is one of the very significant complications of DM2 with a poor prognosis, we decided to use nutritional indicators, such as The Controlling Nutritional Status (CONUT) tool and the Prognostic Nutritional Index (PNI), to check whether patients are well nourished. There are few articles that consider the epidemiology of malnutrition in patients with DM2 based on a small group, and the percentage of patients with poor nutrition differs from 10.6% to 48.2% [7]. A recently published meta-analysis showed that among 18,062 patients, the prevalence of malnutrition was 33% [8]. There are other tools that can be used to indicate malnutrition in patients such as Nutrition Risk Screening (NRS-2002), but we used CONUT and PNI because in these indicators, score calculation is based on objective data, such as laboratory tests [9]. This allowed us to avoid variability in results associated with the subjective opinions of physicians. We had to exclude Subjective Global Assessment (SGA) due to its subjective nature; the retrospective nature of our study meant that we did not have the opportunity to assess the patients analyzed in the study [10]. Due to the same reasons, we had to exclude from our work the Mini Nutritional Assessment (MNA) [11,12].

The aim of this retrospective study was to calculate nutritional indicators in DM2. We used the CONUT and PNI scales to determine the nutritional status of our patients. These indicators require blood test data on albumin level, lymphocyte count and cholesterol level.

## 2. Materials and Methods

The CONUT is a tool for assessing the nutritional status of hospitalized patients based on the results of laboratory blood tests, including total lymphocyte count, cholesterol level and serum albumin concentration. Each parameter is assigned a specific score—lower values result in a higher score and reflect a poorer nutritional status [7,13,14]. Patients can receive between 0 and 6 points for albumin level, 0 and 3 points for lymphocyte count and 0 and 3 points for total cholesterol. The scores for the individual tests are then added together. A total score of 0–1 indicates a normal nutritional status, 2–4 points indicate mild malnutrition, 5–8 points indicate moderate malnutrition and 9–12 points indicate severe malnutrition (Table 1).

The Prognostic Nutritional Index (PNI) is another method of assessing a patient’s nutritional status that relies on just two blood markers: serum albumin and lymphocyte count. The PNI score is calculated using the following formula: 10 × serum albumin (g/dL) + 0.005 × total lymphocyte count (per µL). A low PNI score indicates a higher degree of malnutrition in the patient [14].

These nutritional indicators were chosen because of the objective approach, which excludes observations based on physicians’ experience, as well as possible uncertainties or a thin line in classifying how severe patients’ symptoms are. The ease of use of these tools in medical practice, where efficient and accurate methods are most valuable, increases the probability that physicians will use them to assess the nutritional status of their patients, which can be an invaluable aid in monitoring treatment outcomes and needs.

It must be emphasized that each of these indicators has its limitations, which may affect the accuracy and reliability of the results obtained. In the case of the CONUT index, an important limitation is that it is based on only three laboratory parameters: albumin level, lymphocyte count and total cholesterol level. The PNI index, on the other hand, is based on only two parameters, which makes it a less comprehensive tool.

These parameters can be disturbed not only by malnutrition but also by other factors such as chronic inflammation, infections, liver disease or cancer, which can lead to an overestimation of the values and an incorrect diagnosis of malnutrition. In addition, this index does not take into account anthropometric data such as body weight or BMI, which are important for the overall assessment of nutritional status. Another limitation is the variability in blood test results depending on the patient’s hydration, time of day or clinical condition, which may affect the assessment. In older people, the reliability of the index may be reduced due to frequent coexisting chronic diseases.

In this single-center, retrospective study, we used the database of the Department of Internal, Autoimmune and Metabolic Diseases of the Central Clinical Hospital of the Medical University of Silesia in Katowice from the entire year 2022 from the hospital IT system Asseco Medical Management Solutions (AMMS), which allowed us to record the patient’s medical diagnosis, laboratory test results, medical interview and treatment history. The data were fully anonymized. In accordance with the regulations of the Medical University of Silesia in Katowice, our study is a retrospective study, which does not constitute a medical experiment and for which no evaluation by the Bioethics Committee and no patient consent were required.

### 2.1. Patients

In the period from January 2022 to December 2022, 1600 patients were hospitalized in the Department of Internal, Autoimmune and Metabolic Diseases in the Central Clinical Hospital of the Medical University of Silesia in Katowice. The inclusion criteria were: patients with type 2 diabetes for whom it was possible to calculate the CONUT and the PNI indices. Exclusion criteria included: any other type of diabetes except type 2 diabetes and the absence of measurements required for the calculation of CONUT and PNI. A total of 266 patients were diagnosed with diabetes (24 patients with type 1 diabetes, 242 patients with type 2 diabetes). Of these, 64 patients (35 female and 29 male) met the inclusion criteria and were included in the study.

### 2.2. Analyzed Parameters

Our database included parameters such as: age, sex, weight, height, BMI, body temperature, hemoglobin level, glycated hemoglobin, creatinine, total protein, albumin, natremia, kalemia, cobalamin, folic acid, vitamin D3 level, iron, cholesterol and lymphocyte count.

### 2.3. Tools

We used tools such as Excel and Statistica to collect data, calculate indicators, assess nutritional status and obtain the results. All parameters such as: age, sex, weight, height, BMI, body temperature, hemoglobin level, glycated hemoglobin, creatinine, total protein, albumin, natremia, kalemia, cobalamin, folic acid, vitamin D3 level, iron, cholesterol and lymphocyte count were meticulously entered and organized within the spreadsheet software to create a structured database, and then, using different methods, were analyzed in Statistica.

Prior to conducting comparative and correlational analyses, the distribution of continuous variables was formally assessed using the Shapiro–Wilk test to determine normality. This step was crucial for selecting the most appropriate statistical methods, differentiating between parametric and non-parametric approaches. Differences in continuous variables between two independent groups were analyzed using the independent samples t-test when data followed a normal distribution and the assumptions of the test were met. For data that violated the assumptions of the t-test, the non-parametric Mann–Whitney U test was employed. The t-Student test was applied for comparisons involving PNI scores, likely comparing PNI between groups where parametric assumptions were deemed appropriate or the test was considered robust. Associations between continuous variables were explored using correlation analysis. Pearson’s correlation coefficient (r) was calculated to assess linear relationships between normally distributed variables, while Spearman’s rank correlation coefficient was used for variables that were not normally distributed or were ordinal in nature. Spearman’s test was specifically applied to investigate the correlation between vitamin D levels and nutritional indicators, whereas Pearson’s test was used for other pairwise correlations where appropriate.

## 3. Results

The mean age was 74.9 years with a minimum of 45 and maximum of 95 (SD = 12.33). We classified patients into groups based on their BMI: 27.3% of them had normal weight (BMI 20–24.9), 29.5% were overweight (BMI 25–29.9) and 43.2% were obese (BMI ≥ 30). In Table 2, we present general patients characteristics.

### 3.1. Nutrition Indicators

**Figure 1:**
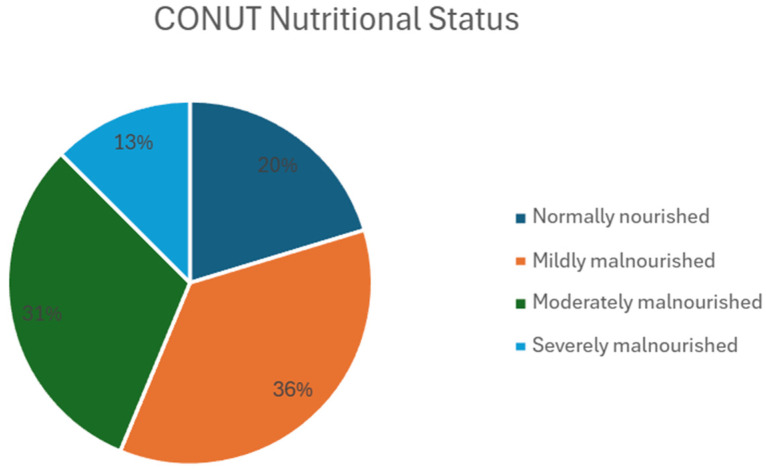
Nutritional status of patients based on CONUT score. Pie chart illustrating the distribution of patients according to their nutritional status, as assessed by the CONUT score. Data are presented for the study cohort (n = 64). The chart shows the percentage of patients classified as normally nourished (n = 13), mildly malnourished (n = 23), moderately malnourished (n = 20) and severely malnourished (n = 8). Abbreviations: CONUT—Controlling Nutritional Status; n—number of patients.

The median CONUT score was 4.00 (range: 0.00–11.00, SD = 3.14). The majority of patients had varying degrees of malnutrition, and only 20.3% were adequately nourished. The largest groups were patients with mild (35.9%) and moderate malnutrition (31.3%), and 12.5% were severely malnourished (Figure 1). Men were more likely to be moderately malnourished, while women were more likely to have mild or severe malnutrition.

**Figure 2:**
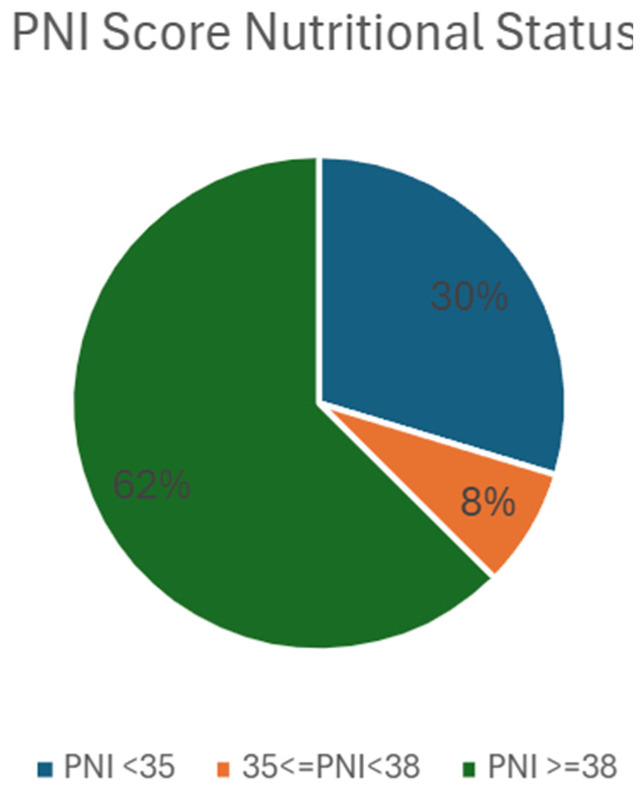
Nutritional status of patients based on PNI score. Pie chart illustrating the distribution of patients according to their nutritional status categories, as defined by the Prognostic Nutritional Index (PNI) score. Data are presented for the study cohort (n = 64). Patients are categorized based on PNI score thresholds: PNI < 35 (poor nutritional status, n = 19, 30%), 35 ≤ PNI < 38 (moderate nutritional risk, n = 5, 8%) and PNI ≥ 38 (good nutritional status, n = 40, 62%). Abbreviations: PNI—Prognostic Nutritional Index; n—number of patients.

The mean PNI was 42.23 (range: 21.05–65.3, SD = 10.11), indicating an overall good protein nutritional reserve in most patients. However, almost 30% of patients had a low PNI, indicating a risk of malnutrition (Figure 2).

There is a very strong, statistically significant negative correlation between the CONUT and PNI values (r = −0.88), which confirms that both scales reflect the nutritional status of the patient’s nutritional status. Vitamin B12 concentration shows a moderate, statistically significant correlation with both CONUT (r = 0.3) and PNI (r = −0.32), indicating an effect of nutritional status on the level of this vitamin. Vitamin D correlated statistically significantly with CONUT (r = −0.34) and weakly statistically insignificantly with PNI (r = 0.29), which could indicate a relationship between vitamin D deficiency and nutritional status. Iron showed a moderate statistically significant correlation with CONUT (r = −0.3) and a weak correlation, statistically insignificant, with PNI (r = 0.21), which could indicate an effect of malnutrition on iron metabolism. Folic acid and HbA1c show only weak or very weak correlations with CONUT and PNI, suggesting that their values are not directly related to the nutritional status of patients. No significant differences were found in CONUT scores based on gender (U-Mann–Whitney test, *p* = 0.1) or in PNI scores (t-Student test, *p* = 0.21). There were no significant correlations between PNI and eGFR, CONUT and eGFR, BMI and CONUT, or BMI and PNI, suggesting that nutritional indices are not strongly associated with renal function or body mass index in this patient group.

### 3.2. Laboratory Tests

In this study, we focused on nutritional indicators, but we also collected and analyzed laboratory tests data. In Table 3, we present laboratory test results (Table 3). We could not calculate the data for all of the patients because not all of them had followed the same laboratory tests. However, for those we could, we decided to include the results in our study.

The average hemoglobin level was 11.47 g/dL, with a minimum of 7.8 g/dL and a maximum of 16.1 g/dL (SD = 2.01). More than half of the patients (57.8%) had anaemia of varying severity, indicating a common problem in the study group. Most patients had mild anaemia (39.1%) and severe anaemia was rare (1.6%). The average lymphocyte count was 1.55 lymphocytes per mm3, with a minimum of 0.16 and a maximum of 4.2 (SD = 0.98). Lymphocytopenia was observed in 55% of patients. The mean total protein level was 5.95 g/dL, with a minimum of 3.89 g/dL and a maximum of 5.15 g/dL (SD = 0.9). The mean albumin level was 3.45 g/dL, with a minimum of 1.54 g/dL and a maximum of 4.69 g/dL (SD = 0.78). Decreased albumin levels were found in 44% of cases. The mean sodium level was 136.71 mmol/L, with a minimum of 119.10 mmol/L and a maximum of 156.00 mmol/L (SD = 4.84): 21 (33%) patients had a decreased sodium level, 42 (66%) had a normal sodium level and 1 (2%) patient had an increased sodium level. The mean potassium level was 4.34 mmol/l, with a minimum of 3.33 and a maximum of 6.34 (SD = 0.59). Hyperkalemia and hypokalemia were relatively uncommon, occurring in 9.4% and 6.3% of patients, respectively. The average total cholesterol level was 146.74 mg/dL, with a minimum of 59.9 and a maximum of 281 (SD = 49.82). Low total cholesterol levels (<100 mg/dL) were found in 14.1% of patients, while 73.4% had normal cholesterol levels (100–200 mg/dL) and 12.5% had elevated levels (>200 mg/dL). The average triglyceride level was 127.23 mg/dL, with a minimum of 40.9 and a maximum of 542 (SD = 73.05). The majority of patients (73.4%) had triglyceride levels below 150 mg/dL, whereas 9.4% had levels exceeding 200 mg/dL. In 63 patients whose creatinine concentration was determined, the mean value was 1.53 mg/dL, with a minimum of 0.47 and a maximum of 10.1 (SD = 1.45). Fifty-nine patients were tested for HDL level: the mean value was 41.87 mg/dL, with a minimum of 10.9 and a maximum of 92.9 (SD = 16.70). Fifty-eight patients were tested for LDL levels: the mean was 78.46 mg/dL, with a minimum of 11.3 and a maximum of 235.2 (SD = 41.46). In 58 patients whose vitamin D levels were determined, the mean value was 24.61 ng/mL, with a minimum of 3.18 and a maximum of 101.0 (SD = 23.18). The glycosylated hemoglobin level was determined in 47 patients: the mean value was 7.01 mmol/mol, with a minimum of 4.79 and a maximum of 12.0 (SD = 1.59). Thirty-three patients were tested for their iron levels. Due to an extremely high SD, we decided to exclude the results of three patients from a group of thirty-six. Exclusion of the outliers was made with the IQR method. The mean value was 48.85 µmol/l, with a minimum of 9.67 and a maximum of 115.0 (SD = 28.95). In 28 patients whose vitamin B12 levels were determined, the mean value was 475.54 pg/mL, with a minimum of 127.0 and a maximum of 1380.0 (SD = 350.38). Twenty-five patients were tested for folic acid levels and the mean was 10.63 ng/mL, with a minimum of 1.15 and a maximum of 84.9 (SD = 16.81). We calculated the eGFR of 45 patients: the mean value was 67.66 mL/min, with a minimum of 7.74 and a maximum of 131.01 (SD = 32.40).

## 4. Discussion

Most patients showed varying degrees of malnutrition, and the strong correlation between the CONUT and PNI indices confirms their diagnostic value. Deficiencies in vitamins, particularly B12 and D and iron, may be associated with nutritional status, although their impact appears to be moderate. A moderate statistically significant correlation was found between vitamin D levels and nutritional status (r = −0.34 for CONUT by Spearman test) and a weak correlation for PNI (r = 0.29 by Spearman test), indicating that higher vitamin D levels are associated with better nutrition. Iron levels were available for 33 patients in our dataset, which showed a moderate negative correlation with CONUT (r = −0.3) and a negligible correlation with PNI (r = 0.21). This suggests that lower iron levels were associated with higher levels of malnutrition.

In this study, no significant association was found between nutritional status and gender, which is consistent with the findings of Lana Husam Ayub and Kameran Hassan Ismail [15]. However, previous studies have come to mixed results. Sanz París et al. reported a significant association between female gender and malnutrition, while Oladimeji Adedeji Junaid et al. found that malnutrition was more common in males [16,17].

Diabetes mellitus impairs metabolism, resulting in the loss of fat-free body mass due to several factors that are common in diabetic patients. These include microalbuminuria, a low-protein diet due to the risk of nephropathy and chronic ulcers (e.g., diabetic foot), which contribute to protein loss and increased daily protein requirements.

Anemia is also prevalent among patients with type 2 diabetes mellitus (T2DM). In our study, 69% (44 patients) had low hemoglobin levels, a common finding in T2DM. Anemia in diabetic patients accelerates the decline in glomerular filtration rate (GFR), contributing to chronic kidney disease and increased cardiovascular mortality [18,19].

In the 45 patients for whom we calculated the estimated glomerular filtration rate (eGFR), the mean value was 67.66 mL/min/1.73m^2^. Renal impairment is defined as an eGFR below 60 mL/min/1.73m^2^, suggesting that renal function was not significantly impaired on average in our group. T2DM leads to renal failure, but this is dependent on disease duration and glycemic control. This may be one of the reasons why patients in our group did not suffer from kidney failure. Another point is that the group of patients was small and, in most cases, there was no information about diabetes duration, so it is possible that kidney problems have not yet developed and patients should be monitored regularly. Kidney disease is related to older age, cardiovascular disease, hypertension, obesity and frequent use of medications that can damage the kidneys (non-steroidal anti-inflammatory drugs), which are becoming increasingly popular nowadays [20,21]. Therefore, it is impossible to determine whether the decrease in eGFR was caused by the age of the patients or the presence of T2DM, the simultaneous presence of both, or other factors and comorbidities altogether.

T2DM is a major contributor to malnutrition, especially in the elderly population. In our study, the mean age of patients was 74.9 years, while other studies report an age range of 52.61 to 80 years [15]. A meta-analysis found that, depending on the study and patient population, 5% to 87% of diabetics are at risk of malnutrition. This can be attributed to various mechanisms, including age-related changes in the sense of smell, taste and gastric acidity, as well as medical, social and psychological factors. In addition, autonomic neuropathy can manifest as diarrhea, gastroparesis or enteropathy, which further increases the risk of malnutrition.

## 5. Conclusions

The CONUT and PNI indicators exhibit a very strong inverse correlation—when the CONUT score increases, the PNI score decreases, both signaling greater malnutrition. Conversely, a low CONUT score and a high PNI score indicate good nutritional status. This suggests that these tools are closely related. Additionally, laboratory results such as vitamin B12, vitamin D3 and iron levels are associated with CONUT and PNI scores. This indicates that malnutrition in T2DM patients may contribute to deficiencies in essential blood parameters, potentially leading to complications such as anemia, osteoporosis and nervous system disorders. In conclusion, nutritional indicators like CONUT and PNI are effective tools for monitoring the nutritional status of T2DM patients. They provide valuable insights into the risk of developing complications and can be useful in everyday medical practice for disease management and prevention. Implementing the CONUT or PNI tools into daily practice should be considered in T2DM patients, especially those over 65 years of age, but taking into account the significant limitations of these indices and the influence of various factors on the laboratory data considered.

## 6. Limitations

The study group in our retrospective study consisted of a small cohort of patients from a single department. This was due to the lack of laboratory data required to calculate the CONUT and PNI indices. Another limitation of this study is the lack of subjective clinical assessment. Since this is a retrospective study, we used only objective indices.

## Figures and Tables

**Table 1:** Nourishment classification based on CONUT score.

Nutrition	CONUT Score
Normal nutrition	CONUT: 0–1
Mild malnutrition	CONUT: 2–4
Moderate malnutrition	CONUT: 5–8
Severe malnutrition	CONUT: 9–12

**Table 2:** General characteristics of the study patients (n = 64). Mean values and standard deviations (SD) for age, height, weight and body mass index (BMI) are presented for the entire cohort.

Mean age	74.9 (±12.33)
Mean height	166.83 (±9.31)
Mean weight	81.52 (±16.58)
Mean BMI	29.57 (±5.27)

**Table 3:** Laboratory test results of the study participants. Mean values, standard deviations (SDs) and normal reference ranges for various hematological and biochemical parameters. The number of patients (n) included for each test is indicated.

Tested Parameter	Mean Result	Norm
Mean hemoglobin level n = 64	11.47 g/dL (±2.01)	M: 13.5–16.5 g/dL F: 11.5–15.0 g/dL
Mean lymphocytes level n = 64	1.55 × 10^3^/µL(±0.98)	1.5–3.5 × 10^3^/µL
Mean total protein level n = 64	5.95g/dL (±0.9)	6.6–8.3 g/dL
Mean albumin level n = 64	3.45 g/dL (±0.78)	3.5–5.2 g/dL
Mean sodium level n = 64	136.71 mmol/L (±4.84)	135–145 mmol/L
Mean potassium level n = 64	4.34 mmol/L (±0.59)	3.5–5.1 mmol/L
Mean total cholesterol level n = 64	146.74 mg/dL (±49.82)	<190 mg/dL
Mean triglyceride level n = 64	127.23 mg/dL (±73.05)	<150 mg/dL
Mean creatinine level n = 63	1.53 mg/dL (±1.45)	M: 0.67–1.17 mg/dL F: 0.51–0.95 mg/dL
Mean HDL level n = 59	41.87 mg/dL (±16.70)	-
Mean LDL level n = 58	78.46 mg/dL (±41.46)	<135 mg/dL
Mean vitamin D level n = 58	24.61 ng/mL (±23.18)	30–50 ng/mL
Mean glycated hemoglobin level n = 47	7.01% (±1.59)	4.8–5.9%
Mean iron level n = 33	48.85 µmol/L (±28.95)	33.0–193.0 µmol/L
Mean vitamin B12 level n = 28	475.54 pg/mL (±350.38)	197.0–771.0 pg/mL
Mean folic acid level n = 25	10.63 ng/mL (±16.81)	3.89–26.8 ng/mL

n—number of patients; F—female; M—male.

## Data Availability

The data presented in this study are available upon request from the corresponding author.
